# Diagnostic hysteroscopy with endometrial fundal incision may improve reproductive outcomes in oocyte recipients after implantation failure

**DOI:** 10.5935/1518-0557.20230037

**Published:** 2023

**Authors:** Nikolaos Peitsidis, Ioannis Tsakiridis, Robert Najdecki, Georgios Michos, Foteini Chouliara, Evi Timotheou, Tatiana Chartomatsidou, Apostolos Athanasiadis, Evangelos Papanikolaou

**Affiliations:** 1 Assisting Nature Centre of Reproduction and Genetics, Thessaloniki, Greece; 2 Third Department of Obstetrics and Gynecology, School of Medicine, Faculty of Health Sciences, Aristotle University of Thessaloniki, Greece

**Keywords:** hysteroscopy, oocyte recipients, pregnancy rates, live birth rates, implantation failure, embryotransfer

## Abstract

**Objective:**

This study aimed to investigate whether hysteroscopy plus endometrial fundal incision (EFI) with endoscopic scissors can improve reproductive outcomes in oocyte recipients who have failed in their first egg donation cycle.

**Methods:**

This was a prospective study (2014-2022) conducted in Assisting Nature Centre Reproduction and Genetics, Thessaloniki Greece, IVF Unit. The study population consisted of oocyte recipients with implantation failure in their first embryo transfer (ET) with donor eggs. All the recipients underwent routine evaluation during their early follicular phase, 1-3 months before the start of a new cycle with donor oocytes and were eligible to undergo EFI.

**Results:**

During the study period, 218 egg recipients underwent egg donation; 126 out of 218 oocyte recipients (57.8%) did not achieve a live birth at the 1st ET. 109 of them had surplus embryos cryopreserved and underwent a second ET; 50 women consented for EFI. Both groups were similar in terms of age, years of infertility, duration of estrogen replacement protocol and number of transferred blastocysts (p>0.05). In the EFI group, 60% had normal intrauterine cavity, while 40% had minor anomalies. The pregnancy test was positive in 46% (n=23/50) in the EFI group compared with 27.1% (n=16/59) in the control group (p=0.04). Moreover, live birth rates were higher in the EFI group compared to the control group (38.0% vs. 20.3%; p=0.04).

**Conclusions:**

The findings of our study indicate that in oocyte recipients after implantation failure, diagnostic hysteroscopy plus EFI prior to subsequent ETmay increase pregnancy and live birth rates.

## INTRODUCTION

Oocyte donation’s success is the highest of all the available assisted reproduction treatments (ART); and pregnancy rates achieved with this technique may be as high as 50% (CDC, 2017). However, there are patients who fail even after several egg donation cycles; from a total of 29,892 egg donation cycles carried out worldwide, 14,647 live births were achieved, which means that 51% of cycles failed to result in a live birth (Barri *et al.*, 2014).

From a clinical perspective, the term “implantation failure” refers to two different types of situations: those with no evidence of implantation (no detectable β-hCG production) and those who have evidence of implantation (detectable β-hCG production), but did not proceed beyond the formation of a gestational sac visible upon ultrasonography (Coughlan, 2018). Although chromosomal constitution of an embryo is the main factor of successful implantation, other factors may also prevent implantation. Particularly, endometritis, endocrine abnormalities, thrombophilia, immunologic factors, anatomic factors that are either congenital or acquired, and may contribute to implantation failure; challenges exist in both the diagnosis and treatment of these factors (Taylor & Gomel, 2008).

Hysteroscopy is considered as the ‘gold standard΄ for assessment of the uterine cavity and the accurate diagnosis of endometrial pathology (Riemma *et al.*, 2022). It also provides a chance for concurrently treating uterine pathologies that can potentially cause infertility such as endometrial polyps, submucous fibroid, intra-uterine adhesions, and septate uterus. Furthermore, published data have shown that operative hysteroscopy for intrauterine acquired or congenital “mechanical” infertility factors are associated with higher rates of live births (Mollo *et al.*, 2009; Bosteels *et al.*, 2010). Additionally, endometrial injury gained popularity over the last years for its possible benefit onimplantation rates (Olesen *et al.*, 2019). Several studies have demonstrated favorable effects on implantation rates, especially in women with repeated implantation failure (RIF) via an induced inflammation plausible mechanism (Siristatidis *et al.*, 2017; Vitagliano *et al.*, 2018).

Our team has adopted a technique of endometrial scratching during hysteroscopy with an endoscopic scissor, which provides targeted real time incision to the fundus of the uterus, the most frequent region of implantation (Minami *et al.*, 2003). Additionally, the operator is able to modify the depth of incision and cause enough scratching, while reducing any possibility of randomly scratching other regions of the intrauterine cavity. The aim of this study was to assessthe impact of endometrial fundal incision (EFI) during hysteroscopy on reproductive outcomesin oocyte recipients, after one implantation failure.

## MATERIAL AND METHODS

### Study population

This is a prospective observational study conducted in the Assisting Nature Centre Reproduction and Genetics, Thessaloniki Greece, IVF Unit. The patients were prospectively recruited between 01.2014 and 09.2022. Oocyte recipients were eligible for the study if: (i) their age ranged between 30 and 50 years, (ii) blastocysts’ transfer was offered, (iii) they had implantation failure in their first embryo transfer (ET) with donor eggs, (iv) absence of submucosal fibromas or polyps in ultrasonography, (v) endometrial thickness >7mm and progesterone blood levels <1.5pg/ml the day before progesterone supplementation during hormone replacement treatment (HRT), and (vi) if EFI was performed, only the endoscopic scissors was allowed. Exclusion criteria were: (i) women who had undergone hysteroscopy within 6 months prior to donor oocyte recipient treatment, (ii) women who had undergone any uterine surgery in the past and (iii) free fluid in the endometrial cavity during HRT preparation.

### Hormone replacement treatment protocol

All frozen embryo transfer (FRET) cycles were carried out following the same hormone endometrial preparation protocol. In particular, starting on day-2 of the cycle, if the scan revealed quiet ovaries and the hormone levels of the woman were basal (E2<80pg/ml and Prog<1.5ng/ml), the patient could initiate HRT. Estrogen supplementation was administered in the form of 17-b estradiol for a minimum of 10 days and a maximum of 20 days before progesterone supplementation. We offered in the second day of the cycle 2mg (1x1), then 4mg (1x2) until day 5, 6mg (1x3) for the next 3 days until day 8, and then 8mg (2x2) onwards until the pregnancy test. Between the 10^th^ and 11^th^ day we checked: i) the endometrial thickness by ultrasound and ii) blood levels of progesterone, LH and E2. If endometrial thickness was less than 7mm the therapy was continued for three more days. Once optimal endometrial thickness was achieved (>7mm), daily progesterone, either vaginally 200mg micronized progesterone TDS, or subcutaneously 25mg progesterone BD, was started and ET was scheduled six days later. The levels of βhCG were examined 9 days post ET or 14 days after the initiation of progesterone supplementation.

### Hysteroscopic procedure

All the recipients were offered routine evaluation during their early follicular phase, 1-3 months before the start of a new HRT cycle with donor oocytes. Moreover, patients planned for hysteroscopy started a contraceptive pill on cycle day-3 (drospirenone and ethinylestradiol) or (chlormadinone and ethinylestradiol), in order to achieve better cavity visualization. A vaginoscopic approach hysteroscopy performed from day 6 to 13 of the menstrual cycle. Routine sedation was administered. Briefly, a rigid hysteroscope -Storz Bettochi 4.8mm hysteroscope (continuous flow; 30° forward oblique view) using a 0.9 normal saline was used. Following adequate distension of the uterine cavity, systematic inspection was performed. Two senior reproductive consultants (R.N. and E.P.) performed all the procedures. EFI was performed witha 2mm Wolf endoscopic scissors; it was performed in a single straight line directed from one fallopian ostium to the other. As far as the depth of incision is concerned, the incision was continued within the connective tissue until the appearance of the first myometrial vessels.

### Reproductive outcomes

Only blastocyst transfers were offered to the recipients. The pregnancy rate was defined as the proportion of women with a positive quantitative serum human chorionic gonadotropin test above 10mIU/ml, 9 days after blastocyst transfer. Clinical pregnancy was declared since heart activity was present starting from 7 weeks onwards; while the first trimester miscarriage rate was defined as the proportion of women with pregnancy loss before 14 weeks of gestation. Live birth was defined as the delivery of a live fetus beyond 24 weeks.

### Statistical analysis

The values of the variables are expressed herein as mean (standard deviation - SD), absolute and relative frequencies, when applicable. Between-group differences for continuous variables were assessed using the independent samples’ t-test. Categorical data were analyzed using the Pearson’s Chi square test and the Fisher’s exact test. Statistical significance was defined as *p*<0.05. The SPSS 25.0 statistical software (IBM Corp.; Armonk, NY, USA) was used for data analysis.

### Ethics

The study protocol was approved by the Institutional Review Board of the IVF Unit (Registration Number: 0501201404). Informed consent was obtained from all the participants in the study.

## RESULTS

**Figure 1:**
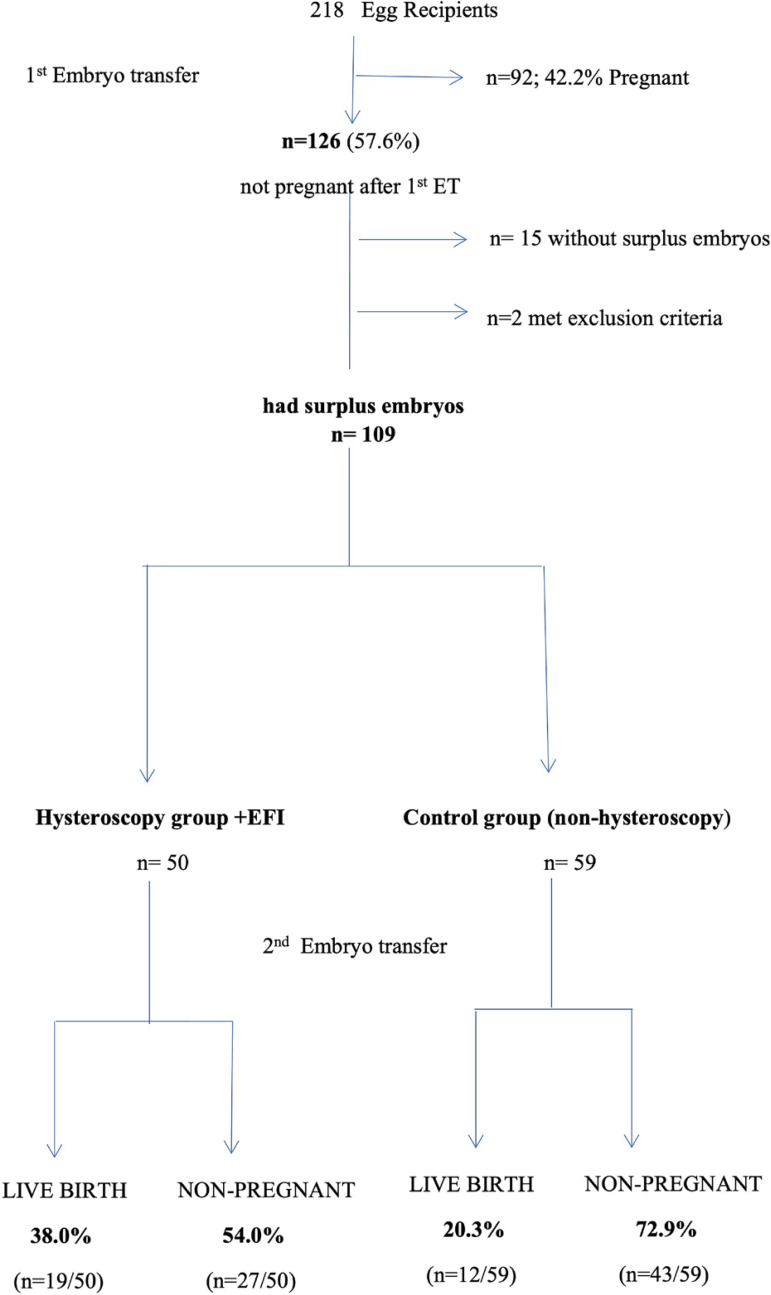
Flowchart of the study population.

During the study period, 218 egg recipients underwent egg donation without hysteroscopy. The rate of those who did not achieve a live birth at the first ET was 57.8% (n=126), while 111 of them had surplus embryos and therefore were eligible for a second FRET. Furthermore, one patient was excluded because she had undergone office hysteroscopy three months prior to her first ET and anotherwoman was excluded due to history of myomectomy four months before her first ET. Both women ended with live birth pregnancies. Finally, 109 egg recipients who had surplus embryos cryopreserved were included and analyzed. These patients underwent double blastocyst ET, unless only one cryopreserved blastocyst was available. In particular, 50 egg recipients underwent EFI with endoscopic scissors, irrespectively of having pathology or not. The other 59 patients, although offered a proper consultation regarding the potential benefits of hysteroscopy, did not consent and were offeredanother ET (Figure 1).

The age of the women included in the study ranged from 35 to 50 years old. The mean duration of infertility was about nine years in both groups. All patients underwent ET with two blastocysts except from ninewomen who preferred single blastocyst transfer to avoid twins and sevenwomenthat did not have enough surplus embryos. The mean blastulation rate was 60.2% in the EFI group, and 59.1% in the control group (*p*>0.05). The demographic characteristics of the participants are shown in Table 1.

**Table 1:** Demographics of recruited patients.

	Hysteroscopy + EFI Group (n=50)	Non-Hysteroscopy Control group (n=59)	*p* value
Age (years ± SD)	40.7 (±6.6)	39.7 (±5.8)	0.4
BMI (kg/m^2^)	28.6 (±4.6)	26.9 (±4.4)	0.05
(%) of patients with history of previous hysteroscopy	3.03% (n=1)	3.38% (n=2)	0.19
Duration of infertility (years)	9.1 (±3.9)	8.5 (±4.4)	0.46
Duration of HRT (days)	17.5 (±1.3)	17.3 (±1.8)	0.51
Mean number of blastocysts transferred	1.7 (±0.5)	1.7 (±0.5)	0.999
Mean total number of blastocysts available for transfer	4.6 (±1.9)	4.6 (±2.0)	0.999
Peak endometrial thickness (mm)	9.7 (±1.5)	10.1 (±1.3)	0.14

# EFI: Endometrial fundal incision, BMI: Body Mass Index, HRT: Hormone replacement treatment.

As for the hysteroscopic findings of the women undergoing EFI, 60% (n=30) of them had normal uterus, 34% (n=17) had partial septate uterus and 2% (n=1) had polyps, endometritis, or adhesions, respectively (Table 2). Regarding reproductive outcomes, the pregnancy test was positive in 46% (n=23/50) in the EFI group compared with 27.1% (n=16/59) in the control group (*p*=0.04). Furthermore, live birth rates were higher in the EFI compared to the control group (38.0% *vs*. 20.3%; *p*=0.04) (Table 3).

**Table 2:** Distribution of hysteroscopic findings in donor oocyte recipients.

Diagnosis	Cases	Percentage
**Normal Uterus**	30	60%
**Polyps**	1	2%
**U1a**	0	0%
**U2a**	17	34%
**U2b**	0	0%
**Endometritis**	1	2%
**Adhesions**	1	2%
**Total**	50	100%

U1a: T shape uterus; U2a: partial septate, arcuate uterus; U2b: septate uterus.

**Table 3:** Reproductive outcomes in the study population.

Outcomes	Hysteroscopy+EFI Group (n=50)	Non-Hysteroscopy Control group (n=59)	*p* value
**Positive β-HCG**	46.0% (n=23)	27.1% (n=16)	***p*=0.04**
**Miscarriage rate <12 weeks**	8% (n=4)	6.8% (n=4)	*p*=0.81
**Live birth rate per ET**	38.0% (n=19)	20.3% (n=12)	***p*=0.04**

#ET: Embryotransfer, EFI: Endometrial fundal incision.

## DISCUSSION

According to the findings of the present study i) hysteroscopy can help in the assessment of the endometrial cavity and the identification of intrauterine pathology and ii) EFI may improve pregnancy and live birth rates in oocyte recipients post implantation failure.

Hysteroscopy remains the gold standard for the evaluation of the uterine cavity, while a great variety of instrumentation enables the performance of the whole hysteroscopic spectrum, from simple diagnostic office procedures without anesthesia to more complicated operative ones (Peitsidis *et al.*, 2023). After the application of ART, intrauterine cavity abnormalities have been proposed as potentially adverse factors affecting pregnancy rates (Peitsidis *et al.*, 2023). Of note, we found about 40% minor intrauterine pathology which is in accordance with previously published data; Cenksoy *et al*. (2013) demonstrated that 44.9% of patients in their study had abnormal hysteroscopic results.

For both patients and clinicians, one of the most discouraging issues in IVF treatment is the recognition of RIF; it has been proven that pregnancy and implantation rates are significantly lower in patients undergoing their second or third cycles of treatment, compared to those undergoing their first cycle of IVF (Bashiri *et al.*, 2018). Dain *et al*. (2014) tried to define the optimal number of failed cycles in oocyte recipients above which the endometrial scratching with Pipelle would show its best effect; no significant benefit of Pipelle scratching was found in women with three, four or five previously failed cycles, but it is worth mentioning that in 79 women with four oocyte donation failures, the Pipelle scratch group showed a clinical pregnancy rate of 31.8%, compared with 14.3% in cycles without Pipelle scratch. This difference did not reach statistical significance (*p*=0.07), but it is clinically important and enhances our results on the possible benefits of endometrial scratching even if they used the different approach of Pipelle. Furthermore, Vitaliano *et al*. (2018) reviewed the evidence on endometrial scratching, finding a benefit when it was performed in patients who had two or more failed ET, but not if patients were undergoing their first cycle. This is also in accordance with our findings, which strengthens the role of endometrial scratching in recipients post implantation failure.

With regards to the possible mechanism of action of endometrial scratching, it mayaffect the expression of endometrial genes involved in the preparation of the endometrium for embryo implantation expression and induce local inflammatory reaction with increased production of cytokines and growth factors, which in turn promote decidual proliferation (Gnainsky *et al.*, 2010). Our approach aimed to increase the mechanical injury of the distension medium by inducing endometrial scratching during hysteroscopy. Hence, we used endoscopic scissors to create EFI in contrast to the Pipelle, where the catheter blindly scratches either the posterior or anterior uterine wall and never the fundus itself. In our technique the injury is directed to the fundus, which is the most common site of implantation after ART, as already mentioned (Minami *et al.*, 2003). The use of endoscopic scissors for endometrial scratching enables a more specific targeted injury only to the fundus of the uterus, while the surgeon can control the depth of the injury. Our method could be easily applied by any reproductive medicine specialist, who routinely carries out hysteroscopies.

To our knowledge, this is the first study determined to examine the influence of EFI during hysteroscopy in oocyte donation recipients post implantation failure after one egg donation cycle. The main strength for this study is the homogeneity in endometrial preparation protocol and embryo quality, since the embryos transferred belong to young women with healthy fertile background, ensuring a limited bias on the pregnancy outcome between groups. Additionally, the complete dataset belonged to a single center and the same two senior reproductive medicine specialists performed all the hysteroscopy and EFI procedures, as this would better overcome possible inter-observer discrepancies. With regards to the initial characteristics, there were no statistically significant differences between the two groups in our study; the body mass index was slightly increased in the intervention group, but even in oocyte donation cycles, increased body mass index contributes to reduced endometrial receptivity, thus strengthening our results (Bellver *et al.*, 2013). A major limitation of this study isthe lack of randomization. Furthermore, in 40% of the participants in the intervention group an intrauterine pathology was identified and treated, which could affect the impact of EFI on the reproductive outcomes. With regards to the group that denied hysteroscopy, the procedure was offered to all women at no extra cost, so it could probably be attributed to the fear of the operation.

## CONCLUSIONS

Many different protocols of endometrial scratching have been proposed in diverse populations undergoing ART, but our study shows for the first time the effect of hysteroscopy and a new proposal of scratching, the so called “EFI”, when performed in the luteal phase prior to ET in egg donor IVF cycles. According to our findings, hysteroscopy with EFI may improve pregnancy and live birth rates in post implantation failure egg donor recipients. More studies, including a higher number of patients with previous implantation failures undergoing egg donor IVF, should be performed in order to evaluate if EFI has an effect in specific subgroups of patients such as thosewith undiagnosed endometrial pathology (septate, arcuate uterus). Furthermore, the additional costs for the patient and the rare adverse effects of hysteroscopy should also be considered, as well as the potential detrimental effects on the endometrium which have not been investigatedas of yet.
